# Dynamic Changes in miR-21 Regulate Right Ventricular Dysfunction in Congenital Heart Disease-Related Pulmonary Arterial Hypertension

**DOI:** 10.3390/cells11030564

**Published:** 2022-02-06

**Authors:** Wei-Ting Chang, Chia-Chun Wu, Yu-Wen Lin, Jhih-Yuan Shih, Zhih-Cherng Chen, Sheng-Nan Wu, Chia-Ching Wu, Chih-Hsin Hsu

**Affiliations:** 1 Division of Cardiology, Department of Internal Medicine, Chi-Mei Medical Center, Tainan 71004, Taiwan; cmcvecho2@gmail.com (W.-T.C.); puupyapple@hotmail.com (Y.-W.L.); s841027@gmail.com (J.-Y.S.); 824zcc@gmail.com (Z.-C.C.); 2 Department of Biotechnology, Southern Taiwan University of Science and Technology, Tainan 71005, Taiwan; 3 Institute of Clinical Medicine, National Cheng Kung University Hospital, College of Medicine, National Cheng Kung University, Tainan 70403, Taiwan; 4 Department of Nephrology, Chi Mei Medical Centre, Tainan 71004, Taiwan; chiachun4481@gmail.com; 5 Department of Pharmacy, Chia Nan University of Pharmacy and Science, Tainan 71710, Taiwan; 6 Department of Health and Nutrition, Chia Nan University of Pharmacy and Science, Tainan 71710, Taiwan; 7 Department of Physiology, National Cheng Kung University Medical College, Tainan 70101, Taiwan; snwu@mail.ncku.edu.tw; 8 Institute of Basic Medical Sciences, College of Medicine, National Cheng Kung University, Tainan 70101, Taiwan; 9 Department of Cell Biology and Anatomy, College of Medicine, National Cheng Kung University, Tainan 70101, Taiwan; 10 Department of Internal Medicine, National Cheng Kung University Hospital, College of Medicine, National Cheng Kung University, Tainan 70403, Taiwan; 11 Department of Internal Medicine, National Cheng Kung University Hospital, Dou-Liou Branch, Yunlin 64042, Taiwan; 12 Department of Respiratory Therapy, College of Medicine, Kaohsiung Medical University, Kaohsiung 807378, Taiwan

**Keywords:** PAH, flow-mediated shear stress, aortovenous fistula, miR-21, RV dysfunction

## Abstract

Right ventricular (RV) failure is a major cause of mortality in pulmonary arterial hypertension (PAH), but its mechanism remains largely unknown. MicroRNA-21 (miR-21) is involved in flow-mediated stress in the vasculature, but its effects on RV remodeling require investigations. Herein, we aim to study the mechanism of miR-21 in the early (compensated) and late (decompensated) phases of PAH-induced RV dysfunction. Using aorto-venous fistula (AVS) surgery, we established a rat model of PAH. To mimic the microenvironment of PAH, we treated cardiomyocytes with flow-mediated shear stress in 6 dyne for 3 and 8 h. To evaluate whether miR-21 could be a biomarker, we prospectively collected the sera of patients with congenital heart disease- (CHD) related PAH. Additionally, clinical, echocardiographic and right heart catheterization information was collected. The primary endpoint was hospitalization for decompensated heart failure (HF). It is of note that, despite an initial increase in miR-21 expression in hypertrophic RV post AVS, miR-21 expression decreased with RV dysfunction thereafter. Likewise, the activation of miR-21 in cardiomyocytes under shear stress at 3 h was downregulated at 6 h. The downregulated miR-21 at the late phase was associated with increased apoptosis in cardiomyocytes while miR-21 mimic rescued it. Among 76 CHD-induced PAH patients, 19 who were hospitalized for heart failure represented with a significantly lower expression of circulating miR-21. Collectively, our study revealed that the upregulation of miR-21 in the early phase (RV hypertrophy) and downregulation in the late phase (RV dysfunction) under PAH triggered a biphasic regulation of cardiac remodeling and cardiomyocyte apoptosis.

## 1. Introduction

Pulmonary arterial hypertension (PAH) is a rare but fatal condition [1,2]. Chronic pressure overload leads to right ventricular (RV) hypertrophy, volume overload and failure [3]. Notably, the survival rate in patients with PAH is closely associated with RV dysfunction [3]. Additionally, different etiologies associated with pulmonary hypertension lead to various outcomes [4]. Apart from other groups, the pulmonary vasculature in patients with congenital heart disease- (CHD) related PAH is continually exposed to hemodynamic forces, including wall shear stresses [5]. However, the impact of flow-mediated stress on RV remodeling remains largely unknown. Most importantly, the major determinant of PAH-induced RV structural and functional maladaptation requires exploration.

MicroRNAs (miRs) are small endogenous noncoding RNAs that regulate the expression of complementary target messenger RNAs [6,7,8]. Dysregulation of microRNAs has been described in various cardiac diseases, including PAH [6,7,9]. Among different signature miRNAs, microRNA-21 (miR-21) is commonly associated with cardiac hypertrophy, heart failure and myocardial infarction [6,10]. In the context of PAH, miR-21 is highly expressed in pulmonary tissue of several PAH rodent models and humans [10,11]. In our previous studies, circulating miR-21 was observed to be positively associated with the severity of RV dysfunction in patients with hypoxia-induced pulmonary hypertension [7,12]. Additionally, in a sheep model of pressure overload-induced pulmonary hypertension, miR-21 is a critical contributor to the development of RV hypertrophy and dysfunction [12]. Nevertheless, given that the stress of PAH toward RV cardiomyocytes is a continuous process, in a lack of sequential observation, the regulatory mechanism of miR-21 on RV remodeling remains uncertain. Herein, through a translational approach, we focused on the dynamic changes in miR-21 expression and the subsequent effects on RV compensation in response to flow-mediated stress overload in PAH.

## 2. Methods

### 2.1. Study Designs of Animals

All animal experimental procedures were approved by the Institutional Animal Care and Use Committee (IACUC; Chi-Mei Medical Center, Tainan, Taiwan) and were performed in accordance with the Guide and Use of Laboratory Animals (Institute of Laboratory Animal Resources). Ten-week-old adult male Sprague–Dawley rats were randomly divided into three groups as follows. (1) Control group: rats received sham operations. (2) PAH early phase group: rats received AVS and studied 7 days after surgery. (3) PAH late phase group: rats received AVS and studied 28 days after surgery. Echocardiography was performed every 7 weeks after surgery. The details of the rat model of AVS and echocardiographic measurements were described in the supplementary materials. After the end of the experiment, the fibrosis and apoptosis in the right ventricle were analyzed by Masson’s trichrome and TUNEL stained, respectively. The levels of miR-21 were measured by quantitative polymerase chain reaction (Table S1). The expressions of apoptosis-associated proteins were measure by western blot. Furthermore, the information regarding histological characteristics and miR-21 in situ can be found in the supplementary materials.

### 2.2. Microflow-Mediated Shear Stress System

The microflow-mediated shear stress system was created by assembling a parallel-plate flow chamber for different types of cells on glass slides. As described previously [13], H9C2 cardiomyocytes were seeded on a glass slide with extracellular matrix coating to form a monolayer and then sandwiched to form a flow channel with a thin silicone gasket. A mixture of 5% CO_2_ and 95% air was constantly supplied at 37 °C during shear stress application. To apply arterial flow, ALSS (6 dyne/cm^2^) was applied for 3 h (early phase) and 8 h (late phase).

### 2.3. Patients and Study Design

In this longitudinal prospective study conducted from 2015 to 2020, we enrolled patients with CHF-induced PAH, including ASD and VSD. According to the European Society of Cardiology/European Respiratory Society (ESC/ERS) guidelines, the diagnosis is defined by findings on right heart catheterization (RHC), including pulmonary arterial pressure ≥ 25 mmHg, pulmonary capillary wedge pressure (PCWP) ≤ 15 mmHg and pulmonary vascular resistance (PVR) > 3 Wood units in the absence of other etiologies such as primary lung disease or chronic thromboembolic pulmonary hypertension [14]. Patients diagnosed with acute right ventricular decompensation were excluded. In addition, we included ten relatively healthy volunteers whose age and sex were matched to our patients with PAH. Blood samples and echocardiographic parameters were collected for comparison. Patients who received surgical or catheter-based closure for more than one year. Each patient provided informed consent. RHC and echocardiography were conducted at diagnosis. The details of RHC and echocardiographic measurements were addressed in supplementary materials. No PAH-specific therapies, including prostacyclin, endothelin receptor antagonist and phosphodiesterase type 5 inhibitor, were prescribed at that time. A functional capacity study including a six-minute walk test was performed prior to RHC. This study was conducted according to the recommendations of the 1975 Declaration of Helsinki on Biomedical Research involving human subjects and was approved by the local ethics committee (IRB: B-ER-106-056). The statistical analysis was described in supplementary materials.

## 3. Results

### 3.1. miR-21 Mediayed Compensation and Decompensation of RV Function in Rats with PAH

**Figure 1:**
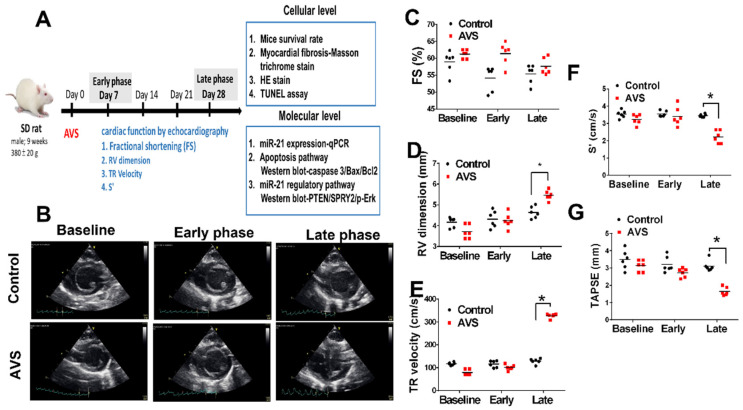
Right ventricular (RV) dysfunction in the late phase of arteriovenous shunt- (AVS) induced pulmonary arterial hypertension (PAH) in rats (**A**) Schematic diagram of AVS-induced PAH in Sprague–Dawley rats in early (7 days) and late (28 days) phases. (**B**) The echocardiography follow-up in rats of the control and AVS groups in early and late phases. Echocardiographic measurements of (**C**) left ventricular fractional shortening (FS), (**D**) RV dimension, (**E**) tricuspid regurgitation (TR) velocity, (**F**) S’ and (**G**) tricuspid annular plane systolic excursion (TAPSE). * *p* < 0.05 for difference between each group. (N = 6–8).

To further investigate the dynamic changes of miR-21 in the progression of PAH, we established a mouse model of PAH by aorto-venous fistula (AVS) and measured the circulating and RV expression of miR-21 in the early (7 days) and late (28 days) phases. To observe the consequences of RV remodeling and dysfunction, we performed serial echocardiography at 7, 14, 21 and 28 days after AVS surgery in rats (Figure 1A,B). Post AVS surgery, there was no significant change in either left ventricular fractional shortening (FS), but there was a significant increase of RV dimension (Figure 1C,D). With an increasing tricuspid regurgitation (TR) velocity in the late phase of rats subjected to AVS (Figure 1E), RV function including RV S’ and TAPSE significantly declined compared with the control group (Figure 1F,G).

**Figure 2:**
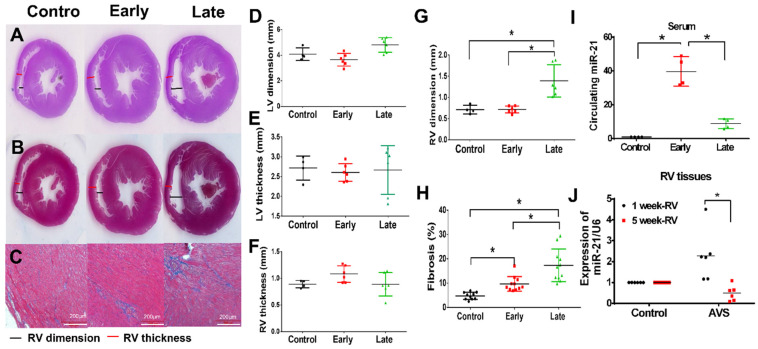
Right ventricular (RV) failure in rats subjected to arteriovenous shunt (AVS) is associated with a downregulation of MiR-21. The rats subjected to AVS in early (7 days) and late (28 days) phases, (**A**) the hematoxylin and eosin stain and (**B**, **C**) Masson trichrome staining of heart sections in indicated groups; scale bars, 200 µm. Quantification of (**D**) LV dimension, (**E**) LV thickness, (**F**) RV thickness, (**G**) RV dimension and (**H**) cardiac fibrosis in indicated groups. The expression of miR-21 in human plasma circulating (**I**) and RV tissues (**J**) in indicated groups. Data are expressed using mean ± standard deviation (S.D.). * *p* < 0.05 for difference between each group. (N = 4–6).

Additionally, the histology of hearts in the early and late phases was measured by HE staining (Figure 2A), and Masson trichrome staining was performed to detect myocardial fibrosis (Figure 2B,C). There was a slight increase in RV thickness at the early phase and significantly enlarged RV dimensions at the late phase, but this increase was not found in the left ventricle in rats subjected to AVS (Figure 2D–G). Additionally, we observed significantly increased fibrosis in the heart tissues of rats subjected to AVS in both the early and late phases (Figure 2H). However, the level of fibrosis in the late phase was markedly higher than that in the early phase. Correspondingly, circulating miR-21 significantly increased in the early phase but dropped in the late phase (Figure 2I). Similar phenomena were also obtained in RV tissues (Figure 2J).

Likewise, the dynamic changes of miR-21 were associated with an upregulation of myocardial injury markers including B-type Natriuretic Peptide (BNP) and A-type Natriuretic Peptide (ANP) but cardiac hypertrophy-related gene myosin heavy chain 7 (MYH7) dropped in late phase of rats subjected to AVS (Figure S1). Collectively, these results indicated that the dynamic changes in miR-21 are associated with compensated RV hypertrophy in the early phase but are associated with decompensated RV dysfunction in the late phase in response to pulmonary hypertension.

### 3.2. The Upregulation of miR-21 in Rats of AVS mainly in RV Cardiomyocytes instead of Fibroblast

To identify the main cells which present high expression of miR-21, using miR-21 oligonucleotide probe we co-stained miR-21 with either F-actin (cardiomyocyte specific) or Vimentin (fibroblast specific). Interestingly, compared with the sham group, there was a significant increase of miR-21 expression in the RV of post-AVS rats. The expression of miR-21 mainly located in cardiomyocytes instead of fibroblasts (Figure S2).

### 3.3. Mir-21 Regulated RV Hypertrophy and Apoptosis in Rats with PAH through the Spry2 and PTEN Pathways

**Figure 3:**
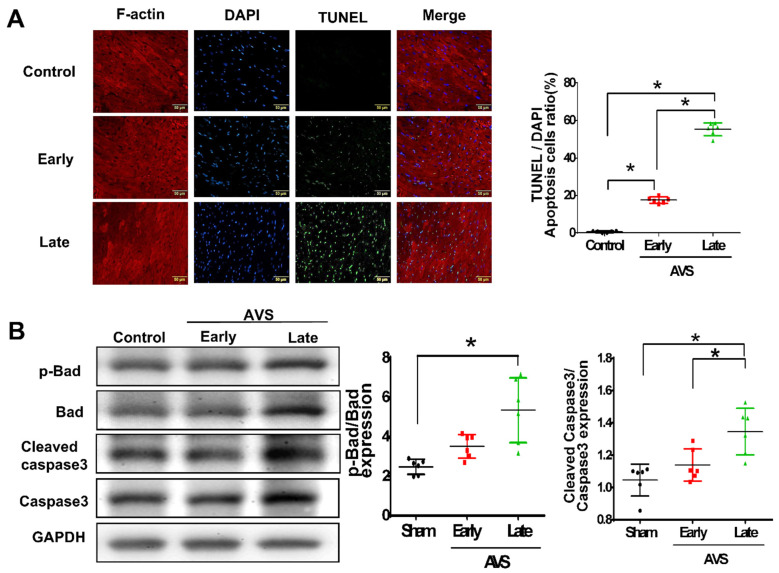
Pulmonary arterial hypertension (PAH) significantly increases apoptosis in right ventricular (RV) cardiomyocytes of rats subjected to arteriovenous shunt (AVS) at the late phase. The post-AVS rats in early (7 days) and late (28 days) phases, (**A**) representative apoptotic cells by terminal deoxynucleotidyl transferase–mediated UTP nick-end labeling (TUNEL) analysis (left panel). Quantification of cardiac apoptosis in indicated groups of rats (right panel). (**B**) Expressions of apoptosis-associated protein were measured by western blot. Representative *p*-Bad/Bad and cleaved caspase 3/caspase 3 ratio in the RV in each group of rats (left panel). The relative expression level of each protein was quantified by densitometry and normalized to the control level (right panel). Data are expressed using mean ± standard deviation (S.D.). * *p* < 0.05, for difference between each group. (N = 6–8).

TUNEL staining showed that the apoptotic cardiomyocytes increased in the RV post-AVS surgery, especially in the late phase (Figure 3A). Alternatively, using western blot, we measured the expression of apoptosis-associated proteins, including cleaved caspase-3 and phosphorylated Bad, while both proteins showed significantly higher expression in the late phase than in the early phase after AVS (Figure 3B).

**Figure 4:**
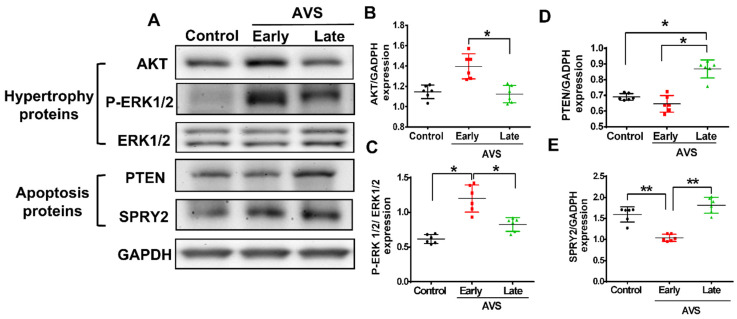
Pulmonary arterial hypertension (PAH) triggers early expressions of hypertrophy-associated proteins and subsequently late expressions of apoptosis-associated proteins in the right ventricle (RV) of rats subjected to arteriovenous shunt (AVS). (**A**) Representative hypertrophy and apoptosis-associated protein in early (7 days) and late (28 days) phase in RV of rat subject to AVS by western blot analysis. Quantification of (**B**) AKT, (**C**) p-ERK, (**D**) PTEN and (**E**) SPRY2 protein expression. Data are expressed using mean ± standard deviation (S.D.). * *p* < 0.05 and ** *p* < 0.01, for difference between each group. (N = 6–8).

Likewise, we also observed that corresponding to the dynamic changes of miR-21, there were slight drops of Sprouty 2 (SPRY2) and tensin homology deleted on chromosome 10 (PTEN) protein expression in the early (compensated) stage but significant increases in the late (decompensated) stage (Figure 4A–C). In contrast, in RV tissues, the expression of AKT and p-ERK, two proteins associated with cardiac hypertrophy, increased in the early phase but declined in the late phase of AVS (Figure 4A,D,E).

### 3.4. Overexpression of miR-21 Mitigates Flow Shear-Induced Apoptosis in Cardiomyocytes

**Figure 5:**
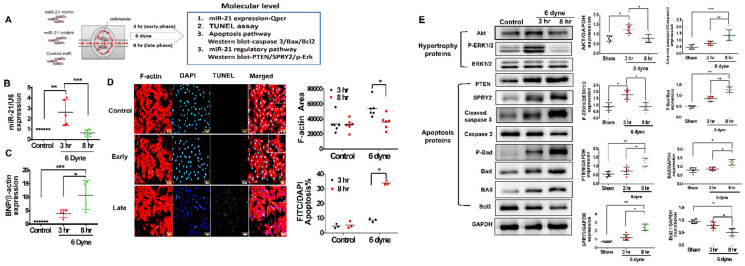
Flow-mediated shear stress triggers a biphasic expression of MiR-21 and is associated with early hypertrophy and later apoptosis-associated protein expressions in cardiomyocytes. (**A**) Schematic diagram of flow mediated shear stress at the early (3 h) and late (8 h) phases at 6 dyne in cardiomyocytes. The relative expression of (**B**) miR-21 and (**C**) B-type Natriuretic Peptide (BNP) was measured by qPCR. (**D**) Immunofluorescence assay of F-actin and terminal deoxynucleotidyl transferase–mediated UTP nick-end labeling (TUNEL) analysis were performed to identify cell area and apoptotic cells, respectively. (**E**) Hypertrophy- and apoptosis-associated proteins in cardiomyocytes were measured by western blot. Data are expressed using mean ± standard deviation (S.D.). * *p* < 0.05, ** *p* < 0.01, and *** *p* < 0.001 for difference between each group. (N = 6–8).

To mimic the environment of volume overload in RV under PAH, we established an in vitro culture system of flow-mediated shear stress (Figure 5A). The expression of miR-21 in cardiomyocytes increased over a short duration (3 h) and decreased over a long duration (8 h) under flow shear stress (Figure 5B). Additionally, the expression of the myocardial injury marker BNP was significantly higher in cardiomyocytes under 6 dynes of flow stress for the long duration of 8 h than for the short duration of 3 h (Figure 5C). Additionally, under flow shear stress at 6 dyne for a long duration (8 h), there was a transient hypertrophic change in the early phase, followed by an increased ratio of apoptosis in cardiomyocytes in the late phase (Figure 5D). Corresponding to the in vivo findings, after a long duration (8 h) of shear stress, the expression of apoptosis-associated proteins, including PTEN, SPRY2, cleaved caspase 3, p-Bad and Bax, increased, whereas the expression of the antiapoptotic protein Bcl2 decreased in cardiomyocytes (Figure 5E). Conversely, AKT and p-ERK decreased in cardiomyocytes stimulated with a long duration (8 h) of shear stress (Figure 5E).

**Figure 6:**
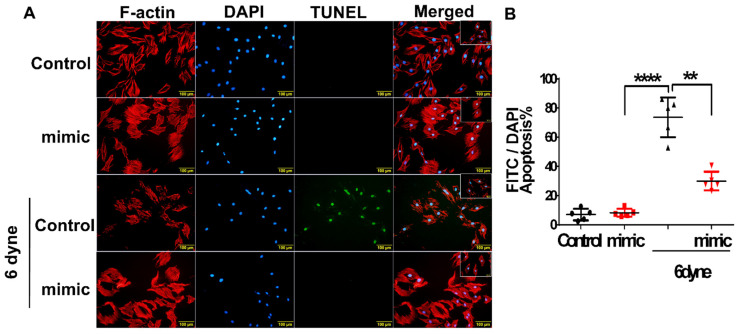
The over-expression of MiR-21 rescues flow shear stress-induced apoptosis in cardiomyocytes. The cardiomyocytes were transfected with miR-21 mimic for 24 h. Cell apoptosis as measured by terminal deoxynucleotidyl transferase-mediated dUTP nick end labeling (TUNEL). (**A**) TUNEL staining (green) indicated cardiomyocytes apoptosis and DAPI staining (blue) indicated cardiomyocyte nuclei. Merged TUNEL and DAPI staining images demonstrated apoptotic cardiomyocyte nuclei. (**B**) Percentage of TUNEL-positive nuclei in each experimental group. Apoptosis rate was calculated as a percent of TUNEL-positive cells out of a total number of cells indicated by DAPI-positive staining for each microscopic field. Data are expressed using mean ± standard deviation (S.D.). ** *p* < 0.01 and **** *p* < 0.001 for difference between each group. (N = 5–7).

To further elucidate our hypothesis that, in the early phase of PAH, shear stress-triggered upregulation of miR-21 plays an antiapoptotic role in maintaining RV function, a miR-21 mimic was used to manipulate the overexpression of miR-21 in cardiomyocytes. In Figure 6, flow shear stress significantly increased the ratio of apoptotic cardiomyocytes for a long duration (8 h), but pretreatment with the miR-21 mimic significantly reduced apoptosis.

**Figure 7:**
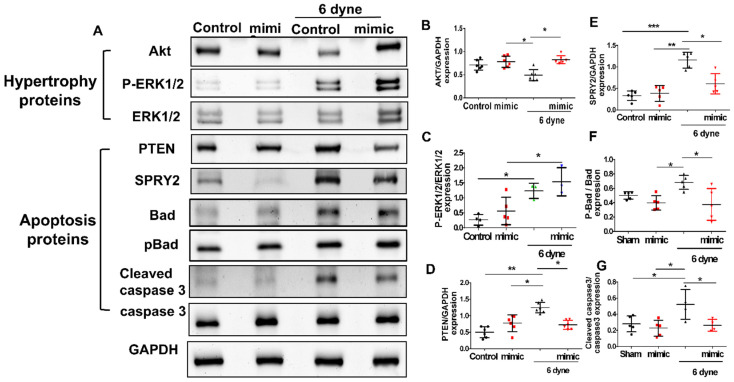
The over-expression of MiR-21 rescues the suppressed expressions of apoptosis-associated proteins in cardiomyocytes under flow-mediated shear stress. The cardiomyocytes were transfected with miR-21 mimic for 24 h. Western blot analysis of hypertrophy- (**A**–**C**) and apoptosis-associate proteins expression and (**D**–**G**) quantification of the results. Data are expressed using mean ± standard deviation (S.D.). * *p* < 0.05, ** *p* < 0.01, and *** *p* < 0.001 for difference between each group. (N = 5–7).

Likewise, as cardiomyocytes were pretreated with the miR-21 mimic, the shear stress-activated expression of PTEN, SPRY2, p-Bad and cleaved caspase 3 decreased, whereas the expression of AKT and p-ERK was increased in cardiomyocytes under shear stress (Figure 7).

**Figure 8:**
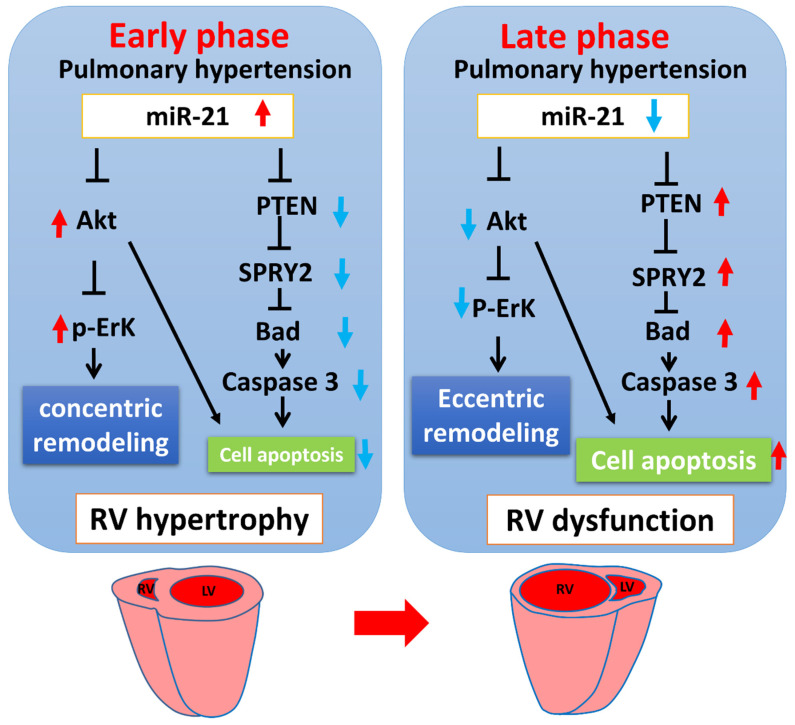
The summary of MiR-21 regulation in the process from RV hypertrophy (early phase) to dysfunction (late phase) under pulmonary hypertension. The up-regulation of miR-21 in the early phase (RV hypertrophy) and down-regulation in the late phase (RV dysfunction) under PAH consequently triggered a biphasic regulation of cardiac remodeling and cell apoptosis.

Interestingly, our findings highlight that under PAH, the upregulation of miR-21 in the early phase (RV hypertrophy) and downregulation in the late phase (RV dysfunction) consequently contribute to the biphasic regulation of cardiac remodeling and cell apoptosis (Figure 8).

### 3.5. Demographic Characteristics of the Enrolled PAH Patients

To further discover the clinical applications of our findings, through an observational study, we aim to investigate whether miR-21 could be a biomarker reflecting RV dysfunction if patients with PAH. Among the 76 patients enrolled in our study, 19 had subsequent hospitalization for heart failure (HF) (Table 1).

The median follow-up duration was 32 months, while the time to event duration was 12 months (IQR: 6, 16 months). There were no significant differences in age, sex or comorbidities, including systemic hypertension and diabetes, between those who did or did not require hospitalization, although we did identify significantly lower body weights among patients who developed HF. In both groups, about one fourth of them had atrial septal defects (ASD), while the others had ventricular septal defects (VSD). More than half of them received either surgical or percutaneous closure of the cardiac shunts. At enrollment, there were no significant differences in the New York functional class (NYFc) or 6MWT between patients with and without decompensated HF. In regard to echocardiographic measurements, although all studied populations had adequate left ventricular ejection fraction (LVEF), patients with PAH showed a dilated right atrial chamber, increased pulmonary arterial pressure and the presence of pericardial effusion compared with the control group. Notably, compared with patients free from hospitalization, those with decompensated HF showed significantly impaired RV function, including tricuspid annular plane systolic excursion (TAPSE) and S’. In terms of RHC, the hemodynamic parameters, including systemic and pulmonary arterial pressures, cardiac index and pulmonary vascular resistance, were similar between the two groups. Notably, despite no specific differences in hemoglobulin or liver and renal function, there was a significant increase in circulating miR-21 in patients with PAH compared with the normal controls. However, among PAH patients who developed decompensated HF, the expression of miR-21 declined (control vs. PAH free from HF vs. PAH with HF as 15.25 ± 6.23 vs. 29.83 ± 37.93 vs. 9.68 ± 21.25, *p* = 0.008). Further, in Cox regression we found that compared with RHC derived mean pulmonary arterial pressure (mPAP), echocardiography derived TAPSE and RV S’, NT-proBNP (HR: 1.12; CI: 1.01–1.28, *p* = 0.05) and circulating miR-21 (HR: 0.92; CI: 0.84–0.99, *p* = 0.02) showed a relatively significant impact on hospitalization for HF in the studied patients. Using the cut-off value of 12, circulating miR-21 remains sensitively associated with hospitalization for HF (HR: 9.62; CI: 1.05–12.16, *p* = 0.04) in the multivariable analysis (Table 2).

Furthermore, in Kaplan-Meier analysis PAH patients with circulating miR-21 less than 12 presented with a significantly higher rate of hospitalization with HF (Figure S3). Taken together, compared with the control subjects, patients with PAH had higher circulating miR-21. However, hospitalization for decompensated HF was associated with not only the development of RV dysfunction but also, interestingly, a decline in miR-21 expression.

## 4. Discussion

PAH is a complex, progressive vascular disease clinically defined as a maladaptive increase in pulmonary arterial pressure in the absence of elevated left heart pressure [3,4,15]. In response to PAH, the RV undergoes structural and functional remodeling, including RV hypertrophy, which is a key determinant of long-term PAH outcomes and is associated with an increased risk of heart failure as well as sudden death [12,15]. However, most previous studies have concentrated predominantly on the impact of biomechanical forces such as shear stress on the pulmonary vasculature. For instance, Happé et al. reported that abnormal pulmonary blood flow could trigger a malignant alteration of pulmonary endothelial cells [16]. Additionally, Nour et al. suggested that intrapulmonary shear stress-mediated endothelial function enhancement could provide an effective target for PAH treatment [17]. Notably, the key determinant of mortality and morbidity of patients with PAH is the consequence of RV dysfunction [15], but the molecular mechanisms driving RV decompensation in the process of PAH remain largely unknown. Hereby, starting from a clinical observation, we found that despite an increasing expression of circulating miR-21 in patients with PAH, there was a significant drop in circulating miR-21 expression along with RV dysfunction among patients who were hospitalized for decompensated HF. Using a rat model of PAH, we also found initial RV hypertrophy followed by subsequent RV dysfunction, accompanied by an early increase but a later decline in miR-21 expression. Furthermore, in a culture system of flow-mediated shear stress, overexpression of miR-21 rescued flow-induced apoptosis in cardiomyocytes. Collectively, our study, for the first time, revealed a dynamic change in miR-21 under PAH-induced shear stress, which could be the turning point of RV decompensation.

According to previous literature, several microRNAs have been observed to be involved in the development of PAH [6,9]. Among them, miR-21 is reported to be highly expressed in pulmonary arterial tissues of several types of human PAH and rodent models [9,12]. Through literature searches, we identified miR-21-targeted genes, including Sprouty 2 (SPRY2) and phosphatase and tensin homology deleted on chromosome 10 (PTEN), which are involved in cardiomyocyte apoptosis [11,15,16]. Green et al. demonstrated that PPARγ ligands regulate proliferative responses by suppressing miR-21 and activating PTEN in pulmonary artery smooth muscle cells post hypoxia [18]. Likewise, through a network bioinformatics approach, Parikh et al. reported that in miR-21-null mice, with the increasing expression of Rho-kinase activity, manifestations of pulmonary hypertension were exaggerated [11]. Under PAH-induced shear stresses, miR-21 has been found to be involved in a positive feedback loop that contributes to the proinflammatory responses of the vascular endothelium [19]. Using a novel anti-miR-21-eluting stent, the post-transplanted arteriosclerosis of coronary allografts was thereafter mitigated [20]. However, the role of miR-21 in cardiomyocytes under overloaded shear stress remains uncertain. Whether it contributes to cardiomyocyte remodeling or dysfunction is the key issue of this study.

In our previous studies, we found that in patients with hypoxia-related pulmonary hypertension, the expression of circulating miR-21 was positively associated with the severity of RV dysfunction [7]. Additionally, in a sheep model of pressure overload-induced pulmonary hypertension, miR-21 could be a key regulator in regional eccentric RV hypertrophy by promoting mitosis but incomplete cytokinesis [12]. Nevertheless, due to a lack of continuous observation, the actual regulatory mechanism of miR-21 on RV remodeling requires emerging evidence. Supporting our previous findings, we found an upregulation of miR-21 at an early stage of PAH, which may contribute to RV hypertrophy, and conversely, the subsequently decreased expression of miR-21 results in RV dysfunction. Mechanistically, we revealed that along with the downregulation of miR-21 at decompensated RV, the expression of SPRY2 and PTEN, regulators of cell apoptosis, increased [21,22]. In contrast, AKT, a regulator of cellular proliferation, and *ERK* phosphorylation, which regulates the balance between eccentric and concentric cardiac growth, were increased [23,24]. Furthermore, using a miR-21 mimic, flow shear stress-mediated apoptosis in cardiomyocytes was mitigated, which provides a potential target for therapeutic interventions.

Our findings indicated that the upregulation of miR-21 in the early phase and downregulation in the late phase may contribute to the initial RV hypertrophy and subsequent dysfunction facing PAH. Since the major contributors to the outcomes of patients with PAH are not only pulmonary arterial pressures but also RV dysfunction [15], the compensatory expression of miR-21 could possibly rescue the failing RV. Given that previous studies have shown that miR-21 mimic-based therapy could be safely delivered to rodents as a novel opportunity to manage obesity and cutaneous wounds [25,26], further in vivo studies focusing on the potentially therapeutic role of miR-21 is crucial. Alternatively, through searching high-throughput chemical screening, we may also discover new modulators of microRNA expressions [27,28]. For example, using small molecule microarrays, inhibitors of miR-21 have been explored [28]. Nevertheless, the regulatory mechanism of miR-21 in crosstalk between RV cardiomyocytes and other cardiac or pulmonary cells should be well studied beforehand. Furthermore, how miR-21 works in other models of PAH, such as hypoxia/sugen, should be investigated.

There are some limitations. Firstly, an optimal mechanism to explain the dynamic changes of miR-21 in RV from the compensatory to de-compensatory phases remains to be explored. Whether the exhaust of endogenous miR-21 contributes to the death of cardiomyocytes requires more evidence. Secondly, although in situ hybridization showed that the up-regulation of miR-21 is mainly located in cardiomyocytes, whether there is crosstalk between cardiomyocytes and fibroblasts remains uncertain. Thirdly, given that there are possibly other microRNAs involved in the flow-overloaded PAH, a more comprehensive approach such as microRNA array may be necessary to identify novel targets other than miR-21. However, upon abovementioned findings, miR-21 could at least be a biomarker for RV failure from maladaptive remodeling and fibrosis to flow-mediated stress.

Collectively, we found that despite an increasing expression of circulating miR-21 in patients with PAH, there was a significant drop in circulating miR-21 expression along with RV dysfunction among patients who were hospitalized for decompensated HF. Using an in vivo rat model of PAH and an in vitro model of flow-mediated shear stress, we also observed a dynamic change in miR-21 in the process of PAH-induced RV dysfunction. By overexpressing miR-21, we, at least in part, mitigated flow-induced apoptosis in cardiomyocytes by suppressing Spry2/PTEN and promoting AKT/ERK phosphorylation. Collectively, our study revealed that the dynamic change in miR-21 could be a key factor determining the timing of RV decompensation under continuous stress from PAH. Further investigations are mandatory to validate our findings.

## Figures and Tables

**Table 1:** The baseline clinical, echocardiographic, functional, hemodynamic and serologic parameters of patients with congenital heart disease-(CHD) related pulmonary arterial hypertension (PAH).

	Normal Control (N = 10)	HF Hospitalization (-) N = 57	HF Hospitalization (+) N = 19	*p* Value
**Clinical parameters**				
**Age (y/o)**	50.2 ± 8.5	52.1 ± 22.2	50.4 ± 23.7	0.64
**Male gender, N (%)**	4 (40)	19 (33.3)	7 (36.8)	
**Body height (cm)**	163.8 ± 17.4	156 ± 25.4	161.6 ± 24.9	0.81
**Body weight (kg)**	68.6 ± 7.1	60.6 ± 15.7	49.5 ± 9.9	0.08
**Diabetes, N (%)**	0	4 (7)	1 (5.2)	0.2
**Systemic HTN, N (%)**	0	9 (15.7)	1 (5.2)	0.61
**Smoking, N (%)**	0	0 (0)	1(5.2)	0.28
**Cancer, N (%)**	0	4 (7)	3 (15.7)	0.36
	**Etiologies**			
**ASD, N (%)**		49 (85.9)	14 (73.6)	0.12
**VSD, N (%)**		8 (14)	4 (21.1)	0.43
**Surgical closure, N (%)**		26 (45.6)	10 (52.6)	0.72
**Percutaneous occluder, N (%)**		6 (10.5)	3 (15.8)	0.81
**Functional capacity**				
**NYFc I, N (%)**	10 (100)	18 (31.6)	4 (21)	0.24
**NYFc II, N (%)**	-	26 (45.6)	9 (47.3)	
**NYFc III, N (%)**	-	13 (22.8)	5 (26.3)	
**NYFc IV, N (%)**	-	0 (0)	1 (5.2)	
**6MWD (m)**	-	404.4 ± 51.1	389.6 ± 82.1	0.68
**Serologic markers**				
**Hemoglobin (mg/dl)**	13.1 ± 2.1	15.5 ± 21.9	12.4 ± 4	0.2
**eGFR (mL/min/1.73m2)**	90.7 ± 38	88.5 ± 44.7	83.9 ± 49.3	0.78
**ALT (IU/l)**	18.9 ± 8.4	24.8 ± 16	25.5 ± 12.1	0.9
**Bilirubin (mg/dl)**	0.9 ± 1.4	0.8 ± 0.3	1.05 ± 0.5	0.39
**NT-proBNP**	12.3 ± 3.8	458.6 ±87.5	613.8 ±61.2	0.01
**Circulating miR-21**	15.25 ± 6.23	29.83 ± 37.93	9.68 ± 21.25	0.008
**Echocardiographic parameters**				
**LVEF (%)**	70.5 ± 6.4	69.5 ± 7.2	72 ± 4.4	0.86
**RA area (cm^2^)**	12.8 ± 4.6	14.9 ± 7.9	15.3 ± 9.7	0.73
**TAPSE (cm)**	2.1 ± 0.5	1.8 ± 0.4	1.1 ± 0.6	0.02
**S’ (cm/s)**	15.6 ± 4.6	11.2 ± 6.7	7.6 ± 5.3	0.04
**PAP (mmHg)**	15.7 ± 2.5	59.6 ± 24.3	72.1 ± 39.3	0.18
**Pericardial effusion, N (%)**	0 (0)	8 (14)	2 (10.5)	0.12
**Right heart catheterization**				
**Heart rate (bpm)**	-	84.7 ± 11.8	86.7 ± 11.1	0.55
**SBP (mmHg)**	-	120 ± 14.7	114.8 ± 9.7	0.18
**DBP (mmHg)**	-	72.1 ± 9.2	70.2 ± 7.3	0.46
**SaO2 (%)**	-	97.2 ± 2.6	98.2 ± 2.2	0.58
**RA pressure (mmHg)**	-	9.2 ± 3.2	11.5 ± 4.4	0.15
**mRV pressure(mmHg)**	-	29.6 ± 10.9	36.2 ± 10.1	0.14
**mPA pressure (mmHg)**	-	39.1 ± 17.1	50.6 ± 13.4	0.08
**Wedge (mmHg)**	-	11.7 ± 3.1	13.7 ± 2.3	0.31
**Cardiac index (l/m^2^)**	-	3.5 ± 0.9	2.6 ± 1.2	0.1
**PVR (woods)**	-	6.1 ± 5.2	8.1 ± 4.5	0.32

Normally distributed parameters are expressed as mean ± standard deviation. Non-normally distributed parameters are expressed as the medians and interquartile ranges. HTN = hypertension; ASD = atrial septal defect; VSD = ventricular septal defect; NYFc =New York functional class; 6MWD = six minute walk distance; eGFR = estimated Glomerular filtration rate is calculated by MDRD equation; ALT = alanine aminotransferase; NT-proBNP = N-terminal pro-brain natriuretic peptide; LVEF = left ventricular ejection fraction; RA = right atrium; TAPSE = Tricuspid annular plane systolic excursion; S’ = Tissue Doppler tricuspid annulus velocity; PAP = pulmonary arterial pressure; SBP = systolic blood pressure; DBP = diastolic blood pressure; RV = right ventricular; PA = pulmonary arterial; PVR = pulmonary vascular resistance.

**Table 2:** The univariate and multivariable Cox regression analysis of hospitalization for heat failure in patients with congenital heart disease- (CHD) related pulmonary arterial hypertension (PAH).

	Univariate		Multivariable			
			Model 1		Model 2	
	HR (95% CI)	*p*	HR (95% CI)	*p*	HR (95% CI)	*p*
Age	0.99 (0.95–1)	0.94				
Male gender	0.57 (0.14–2.1)	0.42				
mPAP (RHC)	1.02 (0.96–1.08)	0.52				
NT-proBNP	1.12 (1.01–1.28)	0.05	1.001 (1–1.02)	0.05	1.001 (1–1.12)	0.08
TAPSE	1.09 (0.24–4.96)	0.9				
RV S’	0.88 (0.61–1.21)	0.52				
Circulating miR-21	0.92 (0.84–0.99)	0.02	0.9 (0.8–0.92)	0.04		
Circulating miR-21< 12	16 (1.92–13.01)	0.01			9.62 (1.05–12.16)	0.01

Abbreviation as Table 1. In Model 1, miR-21 as continuous variables; In Model 2, miR-21 < 12 ng/mL as categorical variables.

## Data Availability

The original data is available upon reasonable request to the corresponding author.

## Supplementary Materials

The following are available online at https://www.mdpi.com/article/10.3390/cells11030564/s1, Figure S1: AVS-induced pulmonary arterial hypertension (PAH) increases myocardial injury in rats, Figure S2: miR-21 in situ hybridization in the RV of sham and rats post AVS, Figure S3: The Kaplan-Meier plot for PAH patients free from hospitalization for heart failure using the cut-off value of 12 of circulating miR-21, Table S1: Primer sequences of rat for real-time RT-PCR. References [7,29,30,31,32,33] are cited in Supplementary Materials.

## Abbreviations and Acronyms

RV	right ventricular
PAH	pulmonary arterial hypertension
miR-21	microRNA-21
HF	heart failure
AVS	Aorto-venous fistula
IPAH	idiopathic PAH
CTD	connective tissue disease
CHD	congenital heart disease
RHC	right heart catheterization
PCWP	pulmonary capillary wedge pressure
PVR	pulmonary vascular resistance
PAP	pulmonary arterial pressure
PCWP	pulmonary capillary wedge pressure
CO	cardiac output
EF	ejection fraction
FS	fractional shortening
