# Supplementary material for: Dynamic Changes in miR-21 Regulate Right Ventricular Dysfunction in Congenital Heart Disease-Related Pulmonary Arterial Hypertension
Source: Cells. 2022 Feb 6;11(3):564. doi: 10.3390/cells11030564 (PMC8834169; doi:10.3390/cells11030564)
Supplement: Supplementary file 1 [file cells-11-00564-s001.zip › cells-1569686-supplementary.pdf]

cells

MDPI

Supplementary Materials

1. Rat Model of AVS

Sprague–Dawley rats (weight 200–250 g) were anesthetized with inhaled isoflurane. Briefly, following a midline abdominal incision, the inferior vena cava (IVC) and aorta were exposed. The aorta was punctured with a 20G disposable needle, and the needle was gradually introduced across the aorta and penetrated the neighboring wall of the IVC.

2. Echocardiography Measurements in Rats

Rats were anesthetized with 1.5% isoflurane and imaged in the supine position, and heart rates were maintained between 400–500 bpm. Using a S6 (General Electric, Boston, MA, USA) echo machine imaging system with a 40-MHz linear probe, a standard 2D echocardiographic study was initially performed in the parasternal long-axis view for assessment of LV dimensions and function [1]. Image depth, width and gain settings were used to optimize image quality. Frame rates were > 150 Hz. By sequential echocardiography, the interventricular septum thickness in diastole (IVSd), left ventricular internal diameter in diastole (LVIDd), LVEF and fractional shortening (FS) were measured.

3. Histopathological Characterization and Mir-21 In Situ Hybridization

After euthanasia, the hearts of rats from each group were excised for histology and molecular analyses. The weight of heart tissue and length of tibia were measured. For histopathological examination, the heart tissue was fixed in 4% paraformaldehyde and embedded in paraffin (Alfa Aesar, Lancashire, UK). The paraffin-embedded sections of mouse hearts stained with hematoxylin and eosin were examined for morphology and hypertrophy as well as Masson's trichrome stained for evaluation of the level of fibrosis. F-actin and Vimentin (Abcam, Cambridge, MA, USA) were stained as the landmark proteins for cardiomyocytes and fibroblast, respectively. The miR-21 *oligonucleotide probe* was designed and purchased from Li-Tzung biotechnology (Kaohsiung, Taiwan). The staining method has been described previously [2,3]. In brief, five-micrometer-thin sections of FFPE tissues adhered to glass slides were deparaffinized in three consecutive xylene, followed by serial dilutions of ethanol and diethyl pyrocarbonate–treated water. Slides were then hybridized in incubation chambers overnight at 37 °C in an oven, using 0.2 µmol/L miR-21 *oligonucleotide* probes. An anti-FITC peroxidase-conjugated antibody (MERCK Millipore, Massachusetts, USA and Sigma-Aldrich Co., St Louis, MO, USA) at 1:100 dilution in TBS/1% bovine serum albumin was applied to the slides for 60 min followed by an anti-FITC antibody conjugated for another 30 min at room temperature.

4. RNA Isolation and Quantitative Real Time-Polymerase Chain Reaction

Total RNA was isolated from myocardium and H9C2 with Trizol (Ambion, Thermo Fisher Scientific, Waltham, MA, USA). cDNA was generated using the Taqman MicroRNA Assays (Foster City, CA, USA). The mRNA level of miR-21 was measured using the 7500 Fast Real-Time PCR system (Applied Biosystems, Foster City, CA, USA). The expression level of miRNA was normalized to GAPDH and U6, respectively. Relative expression levels were calculated with the $2^{-\Delta\Delta Ct}$ method. Primer sequences used in the present study were showed in Table S1.

5. Transfection

After starvation for 6 h, the H9C2 cardiomyocytes were transfected with miR-21 mimic (5′-uagcuuaucagacugauguuga-3′), miR-21 inhibitor (5′-ucaacaucagucugauaagcua-3′) or scrambled control (QIAGEN, Cambridge, MA, USA) at a final concentration of 5 and 20 nM using TransIT-X2® transfection reagent (Mirus, Madison, WI, USA), respectively. At 24 or 48 h after transfection, the cells were harvested for further study.

6. Tunel Assays

Cardiomyocyte apoptosis was quantified by the TdT-mediated dUTP Nick-End Labelling (TUNEL) technique (Promega, Madison, WI, USA). Formalin-fixed sections (10 µm) were treated with proteinase K (20 µg/mL) and then with fluorecein-12-dUTP (50 µm) in the presence of dATP (100 µM) and TdT enzyme at 37 °C. Positive controls were achieved by the treatment of DNase I (1µg/mL, Boehringer Mannheim, Australia). Nuclei were labelled with DAPI (Sigma-Aldrich Co., St. Louis, MO, USA)

7. Western Blot

Equal amounts of proteins were extracted for heart tissues or primary cultured cardiomyocytes and were quantified using the BCA assay (Thermo Fisher Scientific, Waltham, MA, USA). The extracted protein was separated using sodium dodecyl sulfate-polyacrylamide (SDS) gel electrophoresis and transferred onto polyvinylidene fluoride (PVDF) microporous membranes (MERCK Millipore, Burlington, MA, USA). The membrane was blocked with 5% milk and then incubated with antibodies against Caspase 3 (1:500), Bad (1:1000), SPRY2 (1:1000), Erk (1:1000), Akt (1:1000, Cell Signaling, MA, USA), PTEN (1:1000, St Johns, London, UK) or GAPDH (1:3000, Abcam, Cambridge, MA, USA) in TBS buffer at 4 °C overnight. After incubation, the membrane was incubated with horseradish peroxidase-conjugated anti-rabbit/mouse IgG (1:5,000, MERCK Millipore, Burlington, MA, USA and Sigma-Aldrich Co., St Louis, MO, USA) for 1 h at room temperature. Signals were detected using the ECL-Western blotting system (AVEGENE CHEMX 400, Perth, Australia). The intensity of the protein band was quantified by Image J software (Bethesda, Rockville, MD, USA) and the results are expressed as normalized ratio to housekeeping gene GAPDH.

8. Hemodynamic Studies of RHC

In addition to heart rate and blood pressure, data collected from the RHC study included right atrial pressure, systolic, mean and diastolic right ventricular pressures, PAP, PCWP and cardiac output (CO), which were calculated by the Fick method. PVR was calculated using the formula (mPAP-PCWP)/CO.

9. Echocardiography Measurements in Humans

Standard echocardiography was performed (iE33, Philips, Amsterdam, The Netherlands) using a 3.5-MHz multi-phase-array probe in accordance with the recommendations of societies of Echocardiography [4,5]. Left ventricular ejection fraction was measured using the biplane Simpson’s method. The right atrial (RA) area was determined from the apical four-chamber view. PA pressure was calculated according to the combination of RA and RV pressures and the trans-tricuspid flow velocity using the Bernoulli equation. Tricuspid annular plane systolic excursion represents the distance of systolic excursion of the RV annular plane toward the apex. In addition, S’ velocity was measured from the lateral side of the tricuspid annulus using tissue Doppler imaging. All the analyzed images were acquired in three consecutive cardiac cycles and stored digitally at 50–90 frames per second.

10. Human Blood Sampling and Analysis

Blood was collected in EDTA tubes. After centrifugation at 3500 rpm for 15 minutes, sera were stored at −80 °C until analysis. Human plasma biochemical tests (including troponin I and brain natriuretic peptide) and total RNA extraction were performed according to the protocol and prepared for further microRNA real-time PCR [6].

11. Statistical Analysis

Continuous data are presented as the means ± standard deviations or as the medians and interquartile ranges, depending on the nature of the distribution. Dichotomous data are presented as numbers and percentages. Comparisons were evaluated using Student’s t-tests and nonparametric tests (Wilcoxon signed rank test) for continuous variables that were and were not normally distributed, respectively. Chi-square tests or Fisher’s exact tests were used for the categorical variables as appropriate. The Kaplan–Meier method was used to plot MALEs, and group differences were compared via the log-rank test. The hazard ratio (HR) of HF hospitalization was estimated using the Cox regression model. In multivariable analysis, circulating miR-21 was set as a continuous variable in Model 1, while in Model 2, miR-21 < 12 ng/mL was set as a categorical variable. The Statistical Package for the Social Sciences (SPSS) software (version 22.0, IBM SPSS Inc., Chicago, IL, USA) and R (i384, 3.6.1) was used for the statistical analyses.

References

1. Zhu, Z.; Godana, D.; Li, A.; Rodriguez, B.; Gu, C.; Tang, H.; Minshall, R.D.; Huang, W.; Chen, J. Echocardiographic assessment of right ventricular function in experimental pulmonary hypertension. *Pulm. Circ.* **2019**, *9*, 1–9, https://doi.org/10.1177/2045894019841987.
2. Yamamichi, N.; Shimomura, R.; Inada, K.-I.; Sakurai, K.; Haraguchi, T.; Ozaki, Y.; Fujita, S.; Mizutani, T.; Furukawa, C.; Fujishiro, M.; et al. Locked Nucleic Acid In situ Hybridization Analysis of miR-21 Expression during Colorectal Cancer Development. *Clin. Cancer Res.* **2009**, *15*, 4009–4016, https://doi.org/10.1158/1078-0432.ccr-08-3257.
3. Hsu, C.-Y.; Hsieh, T.-H.; Tsai, C.-F.; Tsai, H.-P.; Chen, H.-S.; Chang, Y.; Chuang, H.-Y.; Lee, J.-N.; Hsu, Y.-L.; Tsai, E.-M. miRNA-199a-5p regulates VEGFA in endometrial mesenchymal stem cells and contributes to the pathogenesis of endometriosis. *J. Pathol.* **2013**, *232*, 330–343, https://doi.org/10.1002/path.4295.

4. Rudski, L.G.; Lai, W.W.; Afilalo, J.; Hua, L.; Handschumacher, M.D.; Chandrasekaran, K.; Solomon, S.D.; Louie, E.K.; Schiller, N.B. Guidelines for the echocardiographic assessment of the right heart in adults: A report from the American Society of Echocardiography endorsed by the European Association of Echocardiography, a registered branch of the European Society of Cardiology, and the Canadian Society of Echocardiography. *J. Am. Soc. Echocardiogr.* **2010,** *23*, 685–713, quiz 786–8, doi.org/10.1016/j.echo.2010.05.010
5. Bossone, E.; D'Andrea, A.; D'Alto, M.; Citro, R.; Argiento, P.; Ferrara, F.; Cittadini, A.; Rubenfire, M.; Naeije, R. Echocardiography in Pulmonary Arterial Hypertension: from Diagnosis to Prognosis. *J. Am. Soc. Echocardiogr.* **2013**, *26*, 1–14, https://doi.org/10.1016/j.echo.2012.10.009.
6. Chang, W.-T.; Hsu, C.-H.; Huang, T.-L.; Tsai, Y.-C.; Chiang, C.-Y.; Chen, Z.-C.; Shih, J.-Y. MicroRNA-21 is Associated with the Severity of Right Ventricular Dysfunction in Patients with Hypoxia-Induced Pulmonary Hypertension. *Acta Cardiol. Sin.* **2018**, *34*, 511–517, https://doi.org/10.6515/ACS.201811_34(6).20180613A.

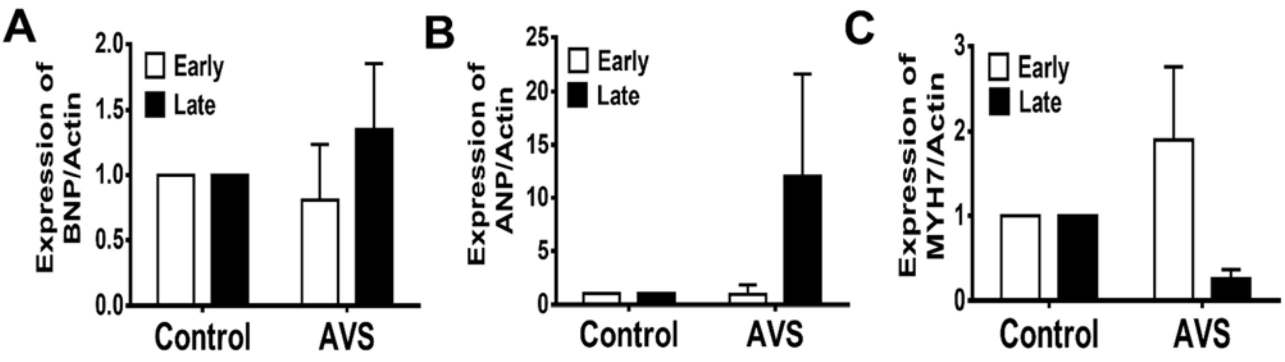

Figure S1. AVS induced pulmonary arterial hypertension (PAH) increases myocardial injury in rats**.** The rats subjected to AVS in early (7 days) and late (28 days) phases, the levels of (**A**) B-type Natriuretic Peptide (BNP), (**B**) A-type Natriuretic Peptide (ANP), and (**C**) myosin heavy chain 7 (MYH7) were measured by qPCR in each groups. *$p < 0.05$ for difference from each group. (n = 6–8).

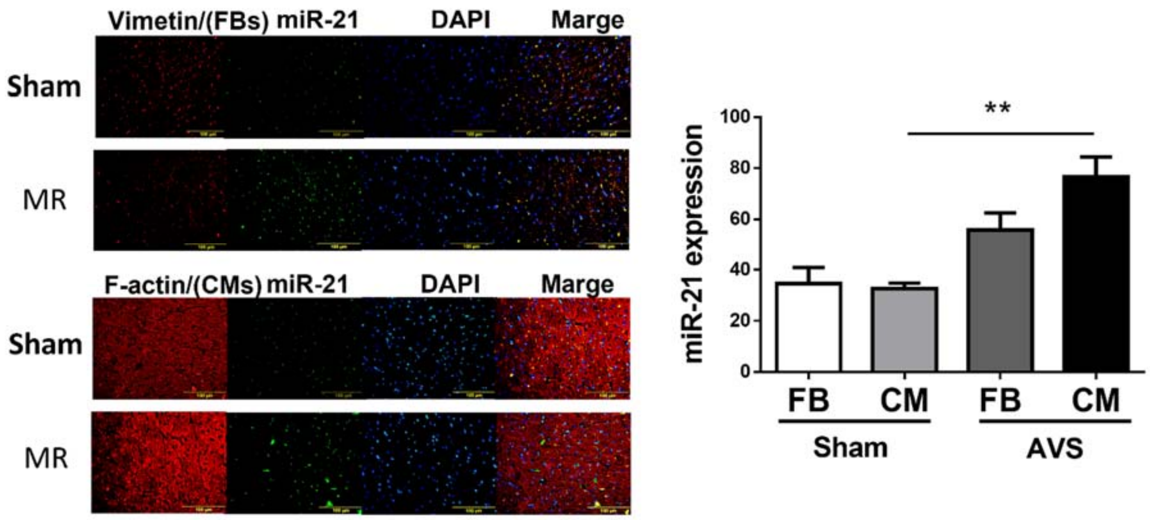

Figure S2. miR-21 in situ hybridization in the RV of sham and rats post AVS. **$p < 0.05$ for difference from each group. (n = 3).

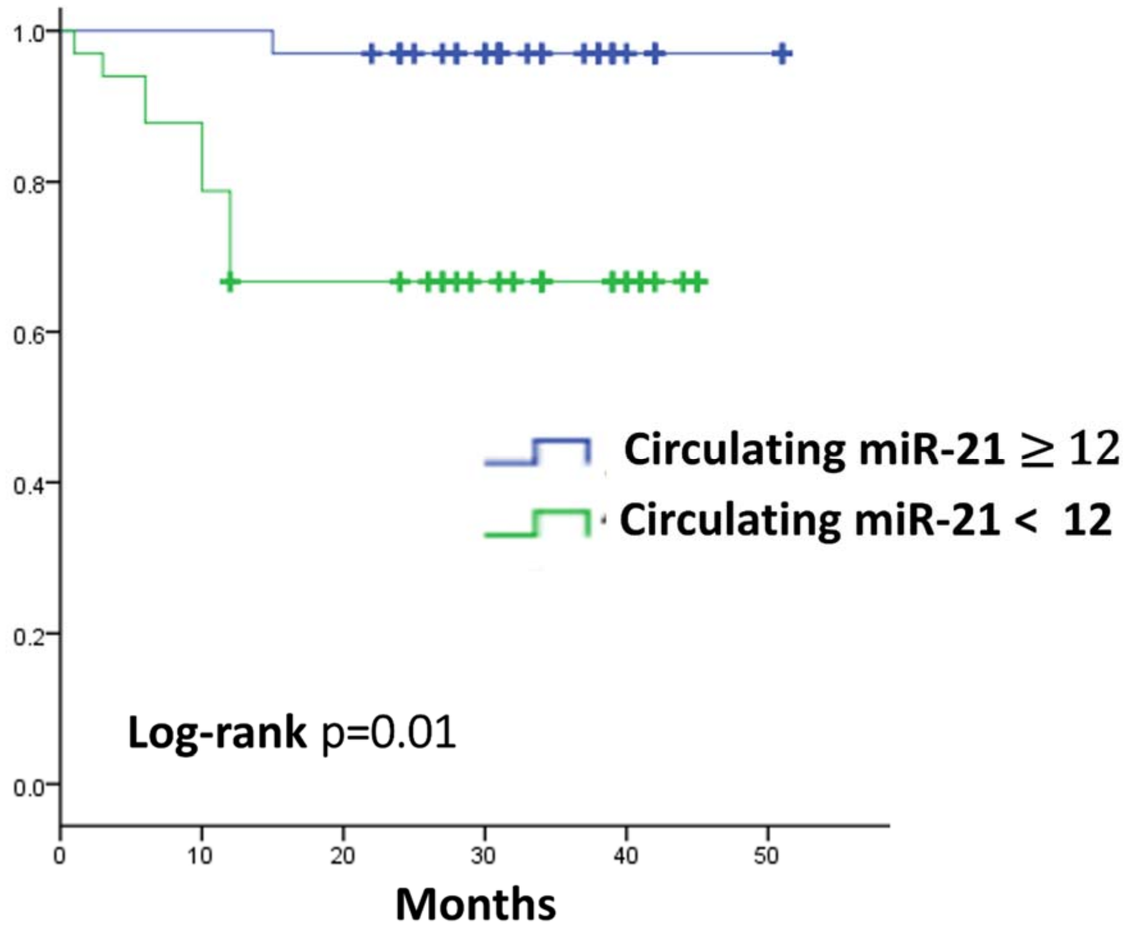

Figure S3. The Kaplan-Meier plot for PAH patients free from hospitalization for heart failure using the cut-off value of 12 of circulating miR-21.

Table S1. Primer sequences of rat for real-time RT-PCR.

Gene	Forward primer	Reverse primer
BNP	GCCAGAACACAACCTGAGCTA	GACAAACAGCAGGTCCAC
ANP	CAACACAGATCTGATGGATTTCA	CCTCATCTTCTACCGGCATC
MyH7	CTGGAGGGTATCCGCATCT	CGGAAGTCCCCATAGAGAATG
GAPDH	TGATTCTACCCACGGCAAGTT	TGATGGGTTTCCCATTGATGA
	**microRNA sequence**	
miR-21	UAGCUUAUCAGACUGAUGUUGA	
